# An Ethnographic Study of Multiple Factors Influencing Perceptions, Attitudes, and Observance of COVID-19 Preventive Measures among Rural and Urban Slum Dwellers in Ghana

**DOI:** 10.1155/2023/1598483

**Published:** 2023-01-31

**Authors:** Matilda Aberese-Ako, Mustapha Immurana, Maxwell Ayindenaba Dalaba, Fidelis E. Y. Anumu, Anthony Ofosu, Margaret Gyapong

**Affiliations:** ^1^Institute of Health Research, University of Health and Allied Sciences, PMB 31, Ho, V/R, Ghana; ^2^Ghana Health Service, Private Mail Bag, Accra, Ghana

## Abstract

**Background:**

The COVID-19 pandemic and government-led interventions to tackle it have had life-changing effects on vulnerable populations, especially rural and urban slum dwellers in developing countries. This ethnographic study explored how the Ghanaian government's management of COVID-19, socio-cultural factors, infrastructural challenges, and poverty influenced community perceptions, attitudes, and observance of COVID-19 prevention measures in Ghana.

**Methods:**

The study employed focused ethnography using in-depth interviews (IDIs), focus group discussions (FGDs), and nonparticipant observations to collect data from an urban slum and a rural community as well as from government officials, from October 2020 to January 2021. The data were triangulated and analyzed thematically with the support of qualitative software NVivo 12. All ethical procedures were followed.

**Results:**

The Ghanaian government's strategy of communicating COVID-19-related information to the public, health-related factors such as health facilities failing to follow standard procedures in testing and tracing persons who came into contact with COVID-19-positive cases, poverty, and lack of social amenities contributed to the poor observance of COVID-19 preventive measures. In addition, the government's relaxation of COVID-19 restrictions, community and family values, beliefs, and misconceptions contributed to the poor observance of COVID-19 preventive measures. Nevertheless, some aspects of the government's intervention measures and support to communities with COVID-19 prevention items, support from nongovernmental organizations (NGOs), and high knowledge of COVID-19 and its devastating effects contributed to positive attitudes and observance of COVID-19 preventive measures.

**Conclusion:**

There is a need for the government to use the existing community structures to engage vulnerable communities so that their concerns are factored into interventions to ensure that appropriate interventions are designed to suit the context. Moreover, the government needs to invest in social amenities in deprived communities. Finally, the government has to be consistent with the information it shares with the public to enhance trust relations.

## 1. Background

Coronavirus disease 2019 (COVID-19) has devastated populations all over the world, with the cumulative number of cases reported globally exceeding 180 million and the number of deaths reaching almost 4 million as of July 2021 [1]. Consequently, the World Health Organisation (WHO) has instituted guidelines on how to prevent, contain, and deal with COVID-19 and, in more recent times, has provided guidelines on the ongoing vaccination programme [2, 3].

Sub-Saharan African countries, like other countries all over the world, have adopted some of WHO's intervention guidelines to prevent and contain the spread of the virus [4]. In Ghana, the initial interventions included partial lockdown in the two most populous cities (the greater Accra and greater Kumasi areas), closure of some public facilities, and a ban on social gatherings. Others were the mandatory use of nose masks in public places, the use of sanitizers, and frequent hand washing with soap, among others [4, 5].

Initially, Ghana was touted as having successfully managed to control the spread of COVID-19, however, the president's address to the nation on 25^th^ July 2021 reported that there was a surge of the delta variant, which was attributed to the disregard for COVID-19 protocols [6]. Consequently, restrictive measures were reintroduced such as no reception should be held during funerals and funerals should last for only two hours, and encouragement of wearing nose masks in public spaces among others [6]. By 15^th^ September 2022, Ghana had recorded a total of 168,616 cases of COVID-19, 167,123 recoveries, 1,459 deaths, and 34 active cases [7]. The question is that why is Ghana still experiencing COVID-19 cases when interventions were put in place at the onset of the pandemic in March 2020? There is therefore an urgent need to understand how intervention efforts are being observed and the factors that are facilitating or hindering progress, especially in vulnerable urban slums and rural communities.

The success of health interventions in responding to pandemics such as Ebola and COVID-19 largely depends on the ability of change agents to work across ethnic, religious, and socio-economic circumstances, which shape community norms, values, belief systems, attitudes, and trust relations [8–11]. In addition, deprived communities such as urban slums and rural communities in developing countries like Ghana face challenges including poor or lack of social amenities and economic opportunities, which might make it difficult for them to observe public health interventions such as COVID-19 preventive measures [12]. For instance, in Ghana, rural and urban slum dwellers work mostly *k* in the informal sector [12]. Thus, beyond their informality, they have low levels of education and less access to basic water and sanitation facilities as well as health facilities, which pose as challenges to their compliance with the preventive measures outlined to contain the spread of the virus and compound their vulnerability.

Various modeling, web-based surveys, and surveys on knowledge, attitudes, and practices regarding COVID-19 preventive measures have been reported [13–17]. A study in Nigeria found that while 60% of respondents believed that COVID-19 was God's punishment, 36% thought it was a man-made virus [18]. Another study in Côte D'Ivoire found that a good number of community members believed that COVID-19 was caused by unnatural factors. Furthermore, the study found that respondents who were educated and those who believed that it was caused by natural events were more likely to observe the COVID-19 preventive measures [19]. Choudhari [17] also noted that internal migrants in India are mostly employed in the informal sector and are marginalized, so they were hard hit by COVID-19 and the government's COVID-19 mitigation measures. So, internal migrants broke the strict lockdown measures and could not adhere to social distancing, since most of them live in crowded quarters that lack social amenities.

Empirical studies carried out among vulnerable informal sector workers in an urban town in Ghana found that residents failed to comply with COVID-19 measures, not because they lacked knowledge on COVID-19, but rather, due to high levels of poverty and lack of infrastructure in the communities to facilitate compliance [20]. Another study carried out in the Cape Coast Metropolis in Ghana, which is also urban, found that knowledge on COVID-19 contributed to improved knowledge on healthcare and healthy living among study participants [21]. Nonetheless, understanding and describing the government's intervention measures and vulnerable populations such as urban slums and rural residents' response is urgently required to inform health policy decision-making. Additionally, due to the nature of COVID-19 and its mitigation measures, the majority of the studies were performed online. This current study contributes to the sparse literature on the behavioral aspects of COVID-19 by employing a focused ethnographic design to explore how the government's intervention measures, socio-cultural factors, poverty, and lack of access to social amenities contributed to community perceptions, attitudes, and observance of COVID-19 preventive measures in a rural and an urban slum in Ghana.

This study makes important conceptual and empirical contributions to the study of COVID-19 through the exploration of how concepts and themes on trust, motivation, demotivation, community values, myths, and misconceptions interact in a natural human environment. Also, it contributes to a critical methodological area of study which is ethnography, which faces ethical and safety challenges in the study of COVID-19, due to its traditional approach to data collection, which is face-to-face interaction with the study subject.

## 2. Methodology

### 2.1. Study Design

The study employed focused ethnography using in-depth interviews (IDIs), focus group discussions (FGDs), and nonparticipant observations to collect data in an urban slum and a rural community from October 2020 to January 2021. An ethnographic qualitative design was the appropriate approach for this study, as it enabled the study team to observe at first-hand interactions in communities and to gain an in-depth understanding of community perceptions, attitudes, and observance of COVID-19 preventive measures [22, 23].

### 2.2. Study Setting

The study settings were an urban slum community in the Oforikrom Municipality of the Ashanti region and a rural community in the Adaklu district of the Volta region of Ghana. The selection of an urban slum municipality in the middle belt and a rural district in the southern belt of Ghana afforded the study two extremes of vulnerability within different contexts in Ghana.

The Oforikrom Municipality is located in the greater Kumasi area of the Ashanti Region, which was an epicenter of COVID-19 at the time of the study [24]. Moreover, the municipality has a large number of internal migrants who are urban dwellers, which offered the study the opportunity to find out how poor and migrant populations complied with the government's COVID-19 mitigation measures. The municipality is located between latitude 6.35°N and 6.40°S and longitude 1.30°W and 1.35°E [25]. The municipality has a population of 213,126, with males and females constituting 107,426 and 105,700, respectively. The economy of the municipality is diverse consisting of trading, service provision, and agriculture such as the cultivation of crops and rearing of livestock [25].

The Volta Region was chosen because it is one of the poorest regions, yet it was ranked number 7 out of the 16 regions in Ghana in terms of the recorded number of COVID-19 cases (626 cases) at the time of data collection in 2020 [24]. Moreover, 23.1% of households in the Volta Region have drinking water quality problems, while 26.6% have no toilet facilities [12]. The Adaklu district in the Volta region, which is rural [26], was chosen to enable the study to explore the experiences of rural communities in dealing with COVID-19. The district is located in the southern part of the Volta Region and lies within longitudes 06°41′1″N and 6.68361°S and latitudes 00°20′1″W and 0.33361°E [27]. It has a population of 38,649, comprising of 18,963 males and 19,686 females [26]. The economy of the district is mainly agrarian; the majority of the residents engage in subsistent farming and livestock rearing and some engage in trading [27].

### 2.3. Selection of Study Communities and Sampling Procedure

The study used a four-stage sampling technique. In the first stage, a purposive sampling technique was used to select the Ashanti and Volta regions. In the second stage, one municipality/district was purposively selected from each region: Oforikrom Municipality in the Ashanti region and Adaklu district in the Volta region. The third stage concerned purposively selecting two communities in each of the selected municipalities/districts for data collection (for the purposes of anonymity, the study communities are referred to as “rural community^*∗*^” and “urban slum^*∗*^”). The fourth stage concerned the selection of study participants. Forty-six IDIs were conducted in the two study sites with public servants, chiefs, elders, persons with disabilities and their caretakers, persons who had tested positive for COVID-19 or whose relatives had tested positive for COVID-19, and community healthcare volunteers (refers to community members who assist healthcare workers in health facilities). Sixty-four people made up of 32 males and 32 females participated in eight FGDs in the two study areas (Table 1).

To understand the multiple factors influencing perceptions, attitudes, and observance of COVID-19 preventive measures in a natural setting, it was important to use a representative purposive sampling technique (maximum variation) [22, 28, 29]. In light of this, the study purposively selected public servants from government institutions who were directly responsible for implementing COVID-19 intervention measures such as the Ghana Health Service (GHS, the service delivery body of the Ministry of Health), Ministry of Gender, and Children and Social Protection, as well as municipal and district assemblies to participate in IDIs. Also, chiefs, queen mothers, and other community leaders (religious leaders and herbalists) were purposively selected to participate in IDIs to represent the community stakeholders' views. Six persons from the two study sites who had ever been diagnosed with COVID-19 or their relative had ever been diagnosed with COVID-19, as well as six persons with disabilities and their caretakers, were purposely selected to participate in IDIs. Since the pandemic was still highly stigmatized at the time of data collection, snowballing was used to select them. Convenience sampling was used to select community members to participate in FGDs to represent the community members' views. FGD participants were informed at least 24 hours before the discussion and community members assisted research assistants (RAs) to mobilize respondents for the FGDs based on age and sex categories (Table 1). Saturation was attained when no new information on the study themes was obtained from the different sampled groups, which is in line with the recommendation [29, 31].

### 2.4. Interview Techniques and Data Collection Process

The research team was made up of a female medical anthropologist (MA) and 8 graduate research assistants (RAs), comprising three females and five males. Four RAs collected data in each study district/municipality. Only RAs who could speak the language(s) commonly spoken in the study areas were recruited. Thus, Dagbani and Hausa languages were for RAs who were recruited to work in the urban slum community in the Oforikrom Municipality and Ewe language for those who were recruited to work in the rural community in the Adaklu district.

The RAs were trained by MA in qualitative data collection methods such as conducting interviews, carrying out observations, and writing observation notes in accordance with Emerson et al.'s [31] recommendation. They were also trained to transcribe interviews.

Four RAs carried out nonparticipant observations in each of the study sites at least one day per week from October 2020 to January 2021. The observations were carried out in public places such as open-air markets, health facilities, funeral grounds, and other community-designated locations, to identify community resources (type of housing, satellite dishes for news feed, toilet facilities, water sources, livelihood sources, and community centers, among others), community functions, availability of hand washing facilities, and how community members were using face masks, and interacting in public. In the Adaklu district, RAs were able to observe and participate in using tippy taps located in front of homes to know whether they contained water and soap.

The IDIs and FGDs sought to understand the following: how communities observed COVID-19 protocols, socio-cultural practices, community values, and perceptions, as well as facilitators and barriers to acceptance and observance of COVID-19 prevention measures (see appendix I and appendix II for study guides and appendix III for observation checklist). IDIs with persons who had ever been infected or had a relative who had ever been infected with COVID-19 enabled a first-hand understanding of experiences of COVID-19 and how it had affected study participants' perceptions and attitudes. IDIs with government officials were aimed at understanding the government's intervention efforts.

IDIs were conducted face-to-face in respondents' chosen location (government workers preferred to be interviewed in their offices, while community leaders and persons who had direct experience with COVID-19 were interviewed mostly in their homes). Persons who were below 18 years old were excluded from the study. Participation was voluntary and a few community members who were not willing to be interviewed were excluded.

In the Ashanti region, interviews were conducted in Hausa and Dagbani, because the study participants are mostly migrants from the northern region of Ghana, whose native language is Dagbani. Hausa is also commonly spoken in the study area by non-Dagbani-speaking migrants. In the Volta region, on the other hand, the study area is made up of an indigenous population, whose native language is Ewe, so community members were interviewed in Ewe. However, in both study sites, public servants were interviewed in English, which is the official language of Ghana. The IDIs took a period of 30 to 35 minutes, while the FGDs lasted an average of one hour and twenty minutes.

A checklist was used to observe the availability of COVID-19 prevention items in homes and public spaces, the daily activities of community members, and how they were observing COVID-19 prevention protocols such as using face masks, social distancing, and hand washing. In addition, smartphones were used to take pictures of some of the critical observations such as social gatherings, and the location of COVID-19 prevention items in public places, and households.

### 2.5. Data Management and Analysis

Interviews were recorded using digital recorders and were transcribed verbatim to maintain responses from study participants. Transcribed data (IDIs, FGDs) and observation notes were uploaded onto a computer and transferred to qualitative software NVivo 12 to support data triangulation, coding, and analysis. Thematic analysis was conducted, which formed the basis for reporting study results.

### 2.6. Ethical Approval and Consent to Participate

The University of Health and Allied Sciences (UHAS) Research Ethics Committee (REC) approved the study, with approval number UHAS-REC A.2 [8] 20-21. Potential study participants were approached and informed of the study and those who were willing to participate were taken through a consenting process. The consent form contained the following information: purpose, procedure, contact details of the principal investigator and the REC administrator, plan to disseminate study findings, date of consent, and signature columns for the interviewer and interviewee. Two copies of the consent form were completed, one was given to the study participant and the second copy was kept by the study team. Participation was voluntary and those who were not interested were automatically excluded, as well as those who were mentally challenged and persons under 18 years old. COVID-19 protocols were observed throughout the study. Interview participants were offered disposable masks and their hands were sanitized before consenting and interviewing. The data sets were anonymized to protect study communities and participants' identities and were accessible to only the study team.

Community entry was carried out in the study communities. The study team visited assembly members, chiefs, and opinion leaders in the study communities. In addition, the district health directorates were visited to inform and seek permission to conduct the study. Permission to conduct the study was granted to the research team by community leaders and government agencies.

## 3. Results

### 3.1. Background of Study Participants

The majority of the study participants in the urban slum were Muslims and had completed basic education, while a few had completed tertiary education. Most of them worked in the informal sector; some of the women were head porters (referred to locally as Kayayei), petty traders, and hairdressers and the men were tailors, scrap dealers, pot makers, drivers, butchers, and some teachers, among others.

The majority of the participants in the rural district were Christians and a few were African traditional believers. Some of the respondents in the rural district had no education, about half of them had completed basic education and a few had completed tertiary education. The majority of the respondents, both men and women were subsistent farmers. Some of the women were petty traders and hairdressers while some of the men were artisans (car mechanics, masons), teachers, and students.

Two major themes were derived from the analysis, which are factors that motivated community members to observe COVID-19 prevention measures and those that demotivated community members from adopting protective measures against COVID-19. The subsequent sections discuss the two major themes.

### 3.2. Motivating Factors to Community Observance of COVID-19 Prevention Measures

All study participants in the IDIs and FGDs indicated that they had heard of COVID-19. Factors that positively influenced community members to practice preventive measures included the government's nationwide intervention measures, provision of COVID-19 prevention items, and support to communities to make appropriate washing aids. Others were individual community members' convictions and knowledge gained from media sources.

### 3.3. Influence of the Media on Positive Attitudes and Observance of COVID-19 Prevention Measures

Some study participants, especially in the urban slum, indicated that their main source of information was the media, which contributed to them observing the prevention measures. Observations in the urban slum community further revealed that most houses hung satellite dishes on the rooftops of their homes, which provided them with access to both local and international news channels (observation notes, urban slum, 30/12/2020). Participants who believed that the virus exists and were observing the recommended protocols indicated that they were influenced by listening to the news on the radio and watching television regularly. They said the media sources gave them ample education and kept them well informed, which influenced how they perceived the virus and protected themselves. Such participants believed that the virus is dangerous and they were convinced that if they protected themselves with the necessary preventive protocols as directed, they were likely to be safe. Such experiences were common among study participants in both the rural community and urban slums.

*“I have been seeing COVID-19 on television and how people are dying, but to be honest, I have never seen someone who was infected with it. I believe it is just a disease and I have never experienced it, but I am protecting myself.”* (IDI, urban slum, community leader 01).

*“I believe it is an airborne disease. I believe it moves in the air for some time and I also believe leaving a distance of 2 meters among people would be helpful in preventing it… Again, I always watch news to be updated…, so I believe 100% that it exists and it is causing a lot of destruction.”* (FGD, men over 30 years, urban slum).

*“I saw how the disease killed dozens of Chinese and how they were dumped and buried … on television, which gripped me with fear, even though none happened in Ghana. I always make sure I practice the safety measures to avert community transmission*.” (FGD, rural community).

Study participants who attested to relying on media sources indicated in IDIs and FGDs that they perceived COVID-19 as a biomedical condition, so anyone could get it. They indicated that such a perception and information from different sources made them to be afraid and increased their willingness to observe COVID-19 prevention protocols. In addition, observations in the study communities revealed that some community members believed that COVID-19 was a normal disease, so they observed the protocols and stated the following in interviews:

*“I believe COVID-19 really exists and I am so scared of it and as a result, I am being cautious,… I do not go outside often.”* (FGD, women over 30 years, urban slum).

*“COVID-19 is a disease we knew nothing about, but it suddenly came to us, so we must protect ourselves well to prevent it from spreading. COVID-19 is a normal disease, which has come to be with us...”* (IDI, assembly man, urban slum).

### 3.4. Motivation to Observe COVID-19 Preventive Measures Based on Government and Nongovernmental Organisations' Support

Study participants indicated that the Ghanaian government's mitigation measures facilitated the observance of COVID-19 preventive measures.

The rural community reported that they received support from the Member of Parliament (MP) for the area, the district assembly, the district directorate of the GHS, and the Ghana Water Company. The district assembly provided “Veronica buckets” (water buckets, which are designed with a pipe at the bottom to facilitate hand washing [32]). The Ghana Water Company provided the community with a water tank and filled it with water regularly at the onset of the pandemic. Observations in the community confirmed the availability of the black plastic water tank with a capacity of about 5000 gallons, which was located in the center of the town (observation notes, rural community, 27/10/2020). Moreover, the district assembly in collaboration with the district directorate of the GHS formed a task force with the assembly members and community leaders in their electoral areas to support communities to observe COVID-19 prevention measures. The MP for the area also donated Veronica buckets to the community and the public healthcare facility located in the community.

Two nongovernmental organizations (NGOs) also supported the community with Veronica buckets in April 2020, which were placed at strategic public places for hand washing. Community members were assigned to always fill them with water, as well as place soap and paper tissue alongside for hand washing. The elders of the community ensured that there was always water in the buckets. The items were picked up at about 10:00 pm each day for safekeeping and were returned the following morning after washing them.

Study participants reported that the NGOs taught them to create locally appropriate hand-washing equipment (commonly referred to as tippy taps), by redesigning plastic gallons into hand-washing containers and water bottles into soap containers. They encouraged each household to own one set or more. Observations carried out in homes revealed that all houses had one or more tippy taps installed in their compounds for hand washing (observation notes, rural community, 16/11/2020).

Study participants reported that the assembly member for the area visited the community about four times during the pandemic (from March to December 2020) to use the information center, which is located in the center of the rural study community (observation notes, rural community, 16/11/2020) to educate the community on COVID-19 and the need to adhere to preventive measures. Also, the task force ensured that the community members did not flout the government's ban on social gatherings. The task force encouraged the community members to report persons visiting the community so that they could ensure that such visitors maintained social distancing. Furthermore, some of the vulnerable people in the rural community received support in the form of money from the government through the Ghana Livelihood Empowerment against Poverty (LEAP) program. They reported that they used the money to buy food and some of the COVID-19 protective items such as soap and face masks. A government official indeed confirmed that the government through the LEAP program offered selected poor and vulnerable households some money to buy food and COVID-19 preventive items:

“*When … COVID-19 came to that height, the LEAP management added something to it* (money) *for them* (poor and vulnerable households)*. … that is what they use in buying the nose masks, soap, and things to be used as a family*.” (IDI, government official, rural community).

In the urban slum, different responses were received to the question on whether the community received support from government agencies and NGOs. The assembly member for the area reported that the community received support from the Municipal Assembly, the GHS, the MP of the constituency, and the National Commission for Civic Education in the form of nose masks and education. Some of the female respondents who work as head potters indicated that an NGO provided the community with COVID-19 prevention items such as Veronica buckets, which were placed in public locations such as mosques for hand washing.

*“The Member of Parliament and the Municipal Chief Executive supported us with nose masks and …, they educated the public about the disease. The National Commission for Civic Education has also supported us by informing the community about the existence of the disease. GHS has also come here* (visited the community) *to inform people about it* (COVID-19) *and people also asked them questions. However, for NGOs, we have not seen some here.”* (IDI, assembly member, urban slum).

The Unit Committee Chairman and most of the men who participated in the FGDs reported that they did not receive any form of support from government officials and NGOs. The FGD participants indicated that they had heard that there was some form of support, however, they were distributed on political party lines, so they were not included. In addition, a healthcare official confirmed that there had been some form of support, but there were complaints that some people did not receive support.

*“I have not seen anyone who has come to support us in this community. Some of us even go to source for funds to come and help the community members.” (*IDI, Unit Committee Chairman, urban slum).

*“I also heard them talking about supporting others. I even saw some giving others rice, money, and other items. However, we those residing around the railway and are in kiosks, we did not receive such support.”* (FGD, males over 30 years, urban slum).

*“Government introduced some packages but we have heard some complaints of not receiving them, so life has really been so difficult.”* (IDI, official, Municipal Health Directorate, urban slum).

Interestingly, some of the urban community members attributed the observance of the COVID-19 preventive measures in their community to the government's security forces that enforced the COVID-19 restrictive measures in their communities.

*“I have realized that there is one reason why we are following preventive measures and that is, God has protected us by allowing the security personnel in this country to monitor people's movements, which resulted in fewer COVID-19 cases recorded*.” (FGD, men over 30 years, urban slum).

### 3.5. Trust in the Government's Mitigation Efforts Influenced Observance of COVID-19 Preventive Measures

Study participants in IDIs and FGDs indicated that they were convinced that the country was at risk of COVID-19 by the recent events in the world, so, they trusted the government and the measures that it had instituted to combat the virus. For such study respondents, even though people tried to discourage them from believing that COVID-19 exists and to dissuade them from observing the preventive measures, they refused to be convinced and practiced the preventive measures. The following quotes illustrate some of the responses:

“*I am so scared about this disease than anybody else. I also believe 100% it exists. You will hear people saying that the government is using it to gain financial support. … which government will agree to take off the cost of water supply, if not because they have no option? Thus, we have to believe the disease exists.”* (FGD, men over 30 years, urban slum).

*“As we have all heard how the disease came about, the advice government has given to us is …since there is no medicine available for now, the hand washing equipment given to us is what we have adopted and we practice hand washing. We have always been on alert and taking good care of ourselves to ensure that we are doing the right thing at the right time*….” (FGD, men over 30 years old, rural community).

### 3.6. Factors Contributing to Negative Attitudes and Practices in the Observance of COVID-19 Preventive Measures

The type of information provided by the government and the attitudes of healthcare providers in healthcare facilities contributed to community members distrusting that the information they received from the government was a true reflection of the magnitude of the virus in Ghana. Moreover, socio-cultural factors such as community values, misconceptions, and myths about COVID-19 contributed to a lackadaisical attitude towards preventive measures. Additionally, poverty, lack of infrastructure, relaxation of the state's intervention measures, and stoppage of support to communities with COVID-19 preventive items hindered the observance of the necessary preventive measures in both communities. The details are elaborated in the subsequent sections.

### 3.7. Government's Approach to Dealing with COVID-19 and Health System Response to COVID-19 Cases Affected the Observance of COVID-19 Preventive Measures

Participants in the rural community indicated in IDIs and FGDs that they believed COVID-19 exists, however, they did not trust the information that the government provided. They argued that the strategy adopted by the government to deal with COVID-19 was not logical. They said that when the numbers of COVID-19 cases were low, the government's intervention measures were strict, yet when the numbers went up, it began to relax the measures. They also argued that the state's timing and approach to COVID-19 intervention suggested that the government was probably misinforming Ghanaians with the motive of exploiting the situation to make money and for political gain.

“*They* (community residents) *believe it exists, only they do not trust the numbers. At first, we were told we were at 100 cases. Then, they* (the government) *said schools should close down. Then, all of a sudden, they said we were at 10,000 going to 15,000 cases. Then, activities resumed, schools reopened, churches resumed, and political activities started. The numbers that they were mentioning were not true… How can Ghana have 20,000 or 22,000 cases and 10,000 will recover in a day? Those are the proofs that they were deceiving us.*” (IDI, healthcare volunteer 01, rural community).

“… *our leaders are using this COVID-19 to seek for money. However, they allow us to go hungry. They are using us. They are lying about it. Even if it was truly here, our weather and the creator did not allow it to spread*.” (IDI, COVID-19 affected 01, urban slum).

Distrust of the government in both urban and rural communities was also influenced by the fact that the advent of COVID-19 in 2020 coincided with party politics toward Ghana's presidential and parliamentary elections, which was scheduled for 7^th^ December 2020. Thus, some of the decisions that were taken by the government were seen as politically motivated, aimed at the incumbent retaining power (re-election). Urban slum residents argued that the government was not keen on strengthening the mitigation measures, so it was not doing much. They contrasted it with the intensity with which the incumbent government's party and other political parties successfully carried out political party rallies and other political programs in communities, yet the government did not use the same strategy and intensity to carry out COVID-19 awareness programs in communities. In the rural area, the study participants argued that the government had used COVID-19 to obtain funds, but had not been fair in the distribution of the funds.

“*We know that the government received a lot of funds from outside, some were to help small-scale businesses. I cannot say all those things are lies, but they were politically motivated. In those days, they were sharing food in Accra, the people I know; be it relatives or others, they did not receive any food…I am not lying...We are in an election year. The vote will depend on how he* (the incumbent president) *worked*.” (IDI, rural community).

In the urban slum, interview participants felt that some of the government's strategies for mitigating COVID-19 and its impact on communities were rather evident that the COVID-19 virus did not exist in the country. One such approach was sharing cooked food with vulnerable populations in poor urban communities that experienced lockdown measures. They argued that a lot of people who were not wearing face masks queued and clamored to collect cooked food, which broke the COVID-19 preventive measures, and yet, most of those people did not contract COVID-19. They argued that if there was COVID-19, then such a measure could have led to a massive spread of the virus. A study participant stated “*It was during the sharing of the food that one could attest to the fact that there is no COVID-19 because everybody would have died*.” (IDI, COVID-19 affected 01, urban slum).

Another source of distrust was the deployment of the military to the urban slum community to enforce the lockdown. The migrants live in wooden kiosks or shacks, which are poorly ventilated, lack pipe-borne water and waste disposals, and have no toilet facilities installed in them (observation notes, urban slum, 10/10/2020). Additionally, some of the residents, especially the males, usually cope with the crowded shacks by converging outside them to interact and access social services, during the day and at night time. These actions resulted in clashes between the slum residents and the military, and some of the residents were assaulted by the military, which resulted in fear and anxiety in the community. It also contributed to community members distrusting the government's mitigation efforts and rather developing the perception that the government was not interested in their welfare. They argued that the government officials and the military who live in regular houses that had all the amenities did not experience the difficulties with their type of resident and thus did not understand them, which motivated the abuse.

“*They* (the *government officials and soldiers) were in their houses, but we were in our kiosks. There was not a thing like, in case there is an issue, we could go to another house to eat and come out. So, the soldiers who sacked us did not try at all. We were in kiosks and we … were tired... so that was the problem they had with us and that is why they were sacking us. We were sitting outside and that was the cause*.” (FGD, males over 30 years, urban slum).

### 3.8. Healthcare Facility Response to COVID-19 Guidelines

Urban slum respondents' personal experiences and encounters with the healthcare system convinced them that the virus did not exist or even if it exists, the government was using it as a ploy to take advantage of the poor and vulnerable. Study participants whose relatives were diagnosed with COVID-19 died in health facilities and were buried by the state expressing their misgivings. They argued that they were not tested for COVID-19 and contact tracing was not done, even though the deceased relatives had lived with people and they had personally been in close contact with them. They added that none of their family members got infected even several months after the death of such relatives. They said these experiences were a testament to the fact that the disease did not exist and that the Ghanaian government was only taking advantage of the vulnerable people in poor communities to solicit for funds. They reported that they did not trust the healthcare system, which they believed was lying about the virus. Such experiences were shared by respondents who lost relatives to COVID-19:

“*For now, I do not believe there is COVID-19, simply because of how we were treated at the hospital. They claimed my mother died due to COVID-19, yet they could not quarantine us, when they know the consequences of being infected with the disease, but until today, we are still alive. When you ask me about my perception of COVID-19, I will say, maybe the government wants to receive some money, and that is why this is happening (government's intervention measures). I took care of my mother and she died six months ago…. Until today (17/10/20), none of us has experienced a headache. “They have put lies on my mother” (the health facility had told lies about his mother). Allah will punish them*.” (IDI, respondent whose mother was diagnosed with COVID-19, urban slum).

*“It was my grandmother who was allegedly infected by the disease* (COVID-19)… *Again, they (hospital staff) did not ask about those who were taking care of her or who had had contact with her for contact tracing and to determine if they were infected,…. I was the one who always bathed and clothed her, so how come I was not infected? For me, I knew they were lying that my grandmother died of COVID-19*.” (IDI, respondent whose grandmother was diagnosed with COVID-19, urban slum).

The practice of isolating persons infected with COVID-19 and ensuring confidentiality was misunderstood by rural community members. Study respondents indicated that they were convinced that COVID-19 did not exist, because they had not been allowed to see and interact with infected persons, who were kept in an isolation center in one of the communities, to enable them to have a first-hand experience of the virus.

“*Nobody can tell me that someone got coronavirus in* “*rural community*.” *They said it, but it was only in writing. It is never true. They took a place at* “*rural community*” *to quarantine people. We wanted to see who had corona. However, they did not give us the opportunity. … If any person really had corona, we wanted to touch that person and allow him to breathe into our faces, so that we know that the person we brought also got corona from the one they claimed was sick… the numbers they were mentioning were fake*.” (IDI, healthcare volunteer, rural community).

Urban slum residents equally argued that they had not seen any persons infected with COVID-19 and such persons had also not been displayed on television, so they did not believe in its existence. Men also believed that women and illiterates had the tendency to doubt the existence of the disease.

“*Frankly speaking, most people within this community have heard about the disease, but I can say about 50% of them do not believe in its existence, because they have not seen an infected person before. Most at times, it is the females and illiterates… who falsify the existence of the disease and they sometimes say that if the disease exists, those who are infected must be shown on television to scare them. However, since they are not seeing such people on the television, it means the disease is not real*.” (FGD, men over 30 years, urban slum).

### 3.9. Influence of Myths and Misconceptions about COVID-19 on the Observance of COVID-19 Preventive Measures

Some of the FGD and IDI participants believed that the African environment is too hot for the virus to thrive, so it does not exist in Ghana. Such study participants felt that they did not have to exert any effort in preventing themselves from contracting COVID-19.

“*People believe that there is heat in Africa, and as a result, the disease does not exist here but rather exists in other continents. The disease cannot stand the heat here*.” (FGD, women over 30 years, urban slum).

“*The whites say the disease is real. Their weather and ours are not the same. See how I am sweating now, there is heat here but abroad is cold. Here, we have diseases that affect the bones, but the whites do not have such diseases. Allah does his things accordingly, so why would they say forcefully it is here? They have snow as their weather... COVID-19 is more like getting a catarrh. It is not like cold weather*. (IDI, respondent whose mother was diagnosed with COVID-19, urban slum).

Study participants in the rural community had the misconception that coughing was a symptom of COVID-19, even without testing for COVID-19. Thus, they believed that anyone who experienced coughing and visited a healthcare facility would be tested for COVID-19, and if the result was positive, the person would be detained and isolated. While more male participants in IDIs and FGDs claimed that women and illiterates believed in such a misconception, the study team's interaction with study respondents revealed that both male and female community members believed in the misconception. This misconception contributed to fear among community members, resulting in the majority of them, both males and females, refusing to seek healthcare in health facilities when they experienced conditions that resulted in coughing. In the urban slum, there was the misconception that COVID-19 was mere catarrh and so it was not as dangerous as citizens were made to believe.

“*Women think it is there, and they fear that they can get it… We heard that if anyone coughing goes to the health facility, they would like to quarantine him or her. So, people do not want to visit the health facility when they are coughing, especially women*.” (IDI, community volunteer 01, rural resident).

“*There was a day when one man here at our mosque said he was infected with the disease and was eventually healed. He said the disease is nothing but mere catarrh. It is Allah who is protecting us*.” (FGD, men above 30 years, urban slum).

Some of the study participants in both the rural community and the urban slum who are Christians, Moslems, and African traditional religion worshippers believed that COVID-19 was a spiritual disease. Some Christians argued that the pandemic is a spiritual disease, which has been caused by the sins of human beings. Additionally, some Muslims believed that everything is destined by the supreme being and for that matter COVID-19 was not necessarily a disease that killed people, but deaths were destined by the supreme being. Such beliefs contributed to a fatalistic approach to the virus and some resorted to invoking divine intervention through prayer and paid little attention to the preventive measures.

“*I think this COVID-19 disease is the outcome of the sins we humans keep committing against God Almighty. We keep doing things which God said we should not* do and that is why this troublesome disease came about.” (IDI, Christian religious leader 02, rural community).

“We, the Muslims so*metimes can put some effort to prevent certain things from happening, but we would not do that and after the worst thing happens, we will say it is God who made it to happen*. “*You are told to protect yourself, you refused, but when you are infected with the disease, you will say it is the will of God*.”” (FGD, men above 30 years, urban slum).

### 3.10. Influence of Community Practices and Values on the Observance of COVID-19 Preventive Measures

Community and family values that the dead should not be spoken ill of contributed to families denying cases of relatives dying from COVID-19. Community members believed that it was derogatory to a dead person and their family members for it to be reported that the person had died from COVID-19-related causes. Consequently, relatives of persons who were suspected to have died of COVID-19-related causes denied such diagnoses and attributed the deaths to pneumonia and other causes, which contributed to community members not believing that COVID-19 was real. This experience was noted in the urban slum, where some respondents had experienced COVID-19 deaths in their families. A respondent in a focus group stated the following:

“*Everybody in the community* (*urban slum*) *has heard about the disease* (COVID-19), *but those who believe in it and are protecting themselves from contracting it will not be up to* 30%... *We have something in common, that is, when one's relative dies as a result of the disease, we refuse to admit that because we see it to be derogatory to the dead person's family. As a result, we then attribute the death to pneumonia, high fever, or other sickness and this does not scare people to believe the disease exists*.” (FGD, men above 30 years, urban slum).

Cultural values of promoting togetherness, close interaction, bonding, and the need to participate in communal activities such as weddings, funerals, and prayers, among others, contributed to the poor observance of the COVID-19 preventive measures. Study participants reported that sometimes they faced pressure and encouragement from community members to flout the COVID-19 preventive measures to participate in social programs. Community members who stuck to preventive measures such as avoiding crowds and using masks at social functions were frowned upon or ostracized.

“*There was a time when I stopped coming for congregational prayers due to the disease, but most of my colleagues here called me a coward*.” (FGD, men above 30 years, urban slum).

In the urban slum, there was constant pressure for community members to flout the COVID-19 protocols due to the need for social bonding. Persons from the same ethnic group perceive themselves as relatives and close friends. They bond through socialization such as communal prayer, attending each other's funerals and weddings, and sometimes eating together, especially for persons who are contemporaries. However, with the advent of COVID-19, some community members chose to eat alone to avoid coming into close contact with others, but they were perceived to be greedy and ostracized. An FGD participant shared his experience:

“*Previously, we used to sit together in the house in the evening to interact or even eat together, but now I eat alone in my room. This has made people to have some hatred for me. Some have stopped talking to me because they think I am isolating myself from them*.” (FGD with men above 30 years, urban slum).

### 3.11. Influence of Poverty on Community Members' Ability to Afford Preventive Measures

It was revealed in interviews as well as during observations in the study communities that, many of the community members did not have resources such as pipe-borne water and waste disposal facilities to facilitate the observance of COVID-19 preventive measures (observation notes, urban slum, 23/11/2020; observation notes, rural community 27/10/2020).

FGDs and IDIs conducted with the study participants also revealed that some could not afford to buy soap to facilitate frequent hand washing, and disposable nose masks that have to be replaced frequently, due to poverty. A nose mask was being sold at 1 GHS (0.18 dollars) at the time of the study, which was beyond the means of most of the respondents. Moreover, respondents explained that they could not afford the cost of a cloth-made nose mask, which was sold between 1 GHS and 2 GHS (0.36 dollars) at the time of the study.

“… *most people here do not wear the mask and the reason is that for some, the money to buy the nose mask is not there. If you want to confirm what I am telling, you just go outside and you will observe that anybody who is passing by is not wearing a mask*... *The provision of nose masks would help to reduce the number of COVID-19 cases*.” (IDI, caregiver of physically challenged 01, urban slum).

“*We need soap, because we are unable to make money to buy soap and that makes us unable to wash our hands regularly*.” (FGD, women below 30 years, rural community).

Government officials from the two study sites further confirmed that community members in their catchment areas were poor and owned only one face mask, which they used for several days, without washing or replacing it. Further observations in both the urban slum and rural community revealed that most of the community members were not using nose masks in public spaces even during public gatherings (observation notes, rural community, 31/10/2020; observation notes, urban slum, 10/10/2020).

Moreover, some of the study participants in the rural community indicated that they could not afford to own television (TV) and radio sets, so they missed out on key information on COVID-19, which was usually passed on to citizens through radio and TV.

### 3.12. How the Relaxation of COVID-19 Protective Measures Influenced Community Attitudes and Behavior towards Prevention

The Ghanaian government started relaxing some of the COVID-19 restrictions such as ending partial lockdown in the two major cities at the end of April, followed by the reopening of schools, among others. Both rural and urban slum dwellers indicated that initially they were observing the protocols, however, they relaxed them after some time, which they attributed to the state's relaxation of COVID-19 preventive restrictions. It was also observed in both study sites that by December 2020, social distancing and wearing of nose masks were no longer being observed by community members. Additionally, by November 2020, most people in the rural community had stopped using the public facilities for hand washing, so the hand washing items were no longer being brought out for communal use.

“*Only the last two weeks the elders packed the things* (Veronica buckets, soap, and tissue, which were donated by the government for hand washing). *They noticed that the hand washing had reduced compared to how it was at the beginning…so there are no more hand washing facilities on the streets*.” (IDI, community volunteer 01, rural community).

The study team further observed that in the third month of the study (December 2020), some of the tippy taps and soap containers located in the homes were empty, they were no longer being filled with water and soap, respectively (observation notes, 14/12/2020, rural community). Both the urban slum and rural dwellers believed that the government relaxed the COVID-19 preventive measures because the virus was no longer in Ghana or had reduced. Furthermore, study participants in the rural community believed that the virus was not prevalent in the rural community, while those from the urban slum perceived that the virus did not exist in their social circles. Such perceptions contributed to the selective application of COVID-19 preventive measures. Study participants explained that they did not use nose masks when they were with their friends and within their communities, but only used them when they were outside their communities, visiting a public facility or when they were in crowded places.

“*I wear my nose mask whenever I am going to a public place like the passport office or license office or even the marketplace*. …*I do not wear a nose mask when I am within* this community, because those I sit and interact with are the people I know. (FGD, men above 30 years, urban slum).

“… It got to the time the president said churches can resume, everything became normal all of a sudden… JHS (Junior High School) students have resumed, as well as SHS (Senior High School) *students*. … *the disease has reduced*… *but still, as for handwashing, we still wash our hands. We still use nose masks when going to crowded places. Only that the way we used to wear it whenever we were going out, with the mind that we may meet someone, that one, a lot of people are no longer doing*.” (rural community, IDI, healthcare volunteer 01).

The study team further observed that the initial enthusiasm with which community members cooperated in establishing opportunities for washing hands with water and soap had reduced, and in some public places and households, such practices had stopped (observation notes, rural community, 14/12/2020). Such changing individual and household attitudes affected the general community attitude, as the provision of water in public places for hand washing had dwindled. The study equally observed that communities were beginning to host large gatherings such as funerals, the inauguration of community development projects, and football matches, among others, and yet the use of nose masks and social distancing were no longer being observed (observation notes, rural community, 19/12/2020; observation notes, urban slum, 04/12/2020).

Additionally, support from the government and NGOs to communities had ceased. For instance, respondents in the rural community indicated that the district assembly had stopped supplying water at the time of the study in November–January, 2021. In the urban slum, there was no longer any form of support from the government. This made it difficult for households to observe the COVID-19 preventive measures.

## 4. Discussion

This focused ethnographic study explored the contextual factors that influenced the perceptions, attitudes, and practices of vulnerable communities in the observance of COVID-19 preventive measures. The study found that the state's intervention measures, government, and NGO support to communities with COVID-19 prevention items and media sources contributed to positive attitudes and observance of the COVID-19 preventive measures. Nevertheless, the government's strategy of communicating COVID-19-related information to the public, poor treatment from health facilities such as health facilities' failure to follow standard procedures in testing and tracing persons who came into contact with COVID-19 diagnosed cases, and poverty and lack of social amenities contributed to the poor observance of COVID-19 preventive measures. Socio-cultural practices, values, and misconceptions also contributed to the poor observance of COVID-19 preventive measures. The state's relaxation of the COVID-19 restrictions and withdrawal of support to communities contributed to the belief that COVID-19 was no longer a problem in the country and this affected the observance of COVID-19 preventive measures.

Generally, the rural community received more support from government sources and NGOs than the urban slum. Support from government and NGOs with items such as Veronica buckets, tippy taps, and water as well as teaching communities to develop locally appropriate tippy taps and containers for soap facilitated the observance of COVID-19 preventive measures in houses and public places. The rural community obtained information on COVID-19 from the GHS, community leaders, the assembly member, and the district task force to keep them well informed on COVID-19. The support helped them to observe the COVID-19 preventive measures. The urban slum received less support probably because, being migrants, most organizations did not perceive the community as their responsibility. Similarly, a study in Saudi Arabia reported that study participants who rated government decisions as effective were more likely to adopt the precautionary measure of practicing hand hygiene compared to those who did not perceive that [33]. Nguyen et al.'s [34] online study among Vietnamese reported that respondents who had access to official COVID-19 information sources and those who were working in healthcare facilities or medical students were more likely to adhere to anti-COVID-19 instructions. Bremmer [35] also found that collaborative efforts and political commitment from different political parties in countries such as Australia and Canada contributed to the successful management of COVID-19.

The government's intervention efforts contributed to some community members believing that the virus exists and ensured trust, which influenced their observation of COVID-19 preventive measures. Lim et al.'s [36] study in Singapore found that citizens trusted the information that was provided by the government and other public sources of information, which promoted adherence. In South Africa, people with higher trust in the government were found to be more likely to engage in positive behavioral change [20]. Faour-Klingbeil et al.'s [14] study in Jordan, Lebanon, and Tunisia found that Jordanians demonstrated more trust in the guidance, the content of the information provided by their local government authorities, and their performance compared to Tunisians and Lebanese's trust in their local governments. This was influenced by the Jordanian government's better management of communication, food, and health risks compared to the governments of Tunisia and Lebanon.

Community members who listened to the radio and watched TV news regularly gained extensive knowledge on the virus, which influenced their attitudes to practice the preventive measures. Additionally, such persons perceived COVID-19 as a biomedical condition, so they believed in the preventive measures and observed them. Similarly, Dryhurst et al. [13] found that people who had ample knowledge of COVID-19 and received information from family members and media such as TV or radio were more likely to perceive risk compared to those who did not have access to such sources. Furthermore, the role of effective communication in getting people to accept and protect themselves against COVID-19 has been noted in another study [37].

The study participants distrusted the government's mitigation efforts. They perceived the government's relaxation of COVID-19 preventive measures after Ghana had started recording high numbers of COVID-19 cases and the sudden report of a high rate of reported COVID-19 recovered cases, as conflicting and illogical. Initially, Ghana was perceived as managing the COVID-19 pandemic well, with the initiation of various mitigation measures. However, the lifting of the COVID-19 measures, which coincided with the rise in party political campaigns, contributed to distrust of the government's commitment to fighting the pandemic. It is not surprising that Asiedu et al. [38] found that trust in the Ghanaian government, which was high at the onset of the pandemic, decreased from 82% in April to 72% in August 2020. Such contradictions affected the study respondents' attitudes toward COVID-19 prevention measures. Similarly, studies carried out in Europe and North America found that study respondents who did not trust government information sources were less likely to adhere to COVID-19 prevention protocols [39].

The government adopting a one-size-fits-all measure of lockdown for the two most populous cities in Ghana without taking into account the context that the residents lived in made the observance of COVID-19 challenging for urban slum dwellers. The urban slum dwellers have limited access to public amenities such as piped-borne water, toilets, good housing, and healthcare facilities, among others, which made it difficult for them to observe the necessary COVID-19 protocols. Deploying soldiers and forcing community members to stay indoors despite lacking basic amenities in the urban slum contributed to the feeling of frustration, skepticism, and loss of trust in the government's mitigation efforts. A study in Ethiopia found skepticism and mistrust among the Oromia and Amhara people towards the government in general and its COVID-19 policy. Such outcomes were influenced by a perceived lack of genuine political representation coupled with the heavy presence of law enforcement units and the military in their communities [40]. Similarly, Choudhari [17] found that internal migrants in India suffered from isolation, poor housing, and lack of access to healthcare services. They were engaged in the informal sector, so the lockdown measures contributed to the lack of access to food and difficulty in the observance of social distancing because they lived in crowded quarters. Guadagno [41] explains that migrants' specific patterns of vulnerability often lie at the intersection of class, race, and status. Migrants are over-represented in low-income and discriminated minorities and encounter unique sets of challenges linked to their lack of entitlement to healthcare, exclusion from welfare programs, and fear of stigmatization.

Failure of healthcare facilities to carry out the recommended testing and contact tracing of persons who came into contact with those diagnosed with COVID-19 contributed to community members' distrust of the healthcare system and the government's intervention efforts. The fact that no other family members were tested and none showed any signs of having contracted COVID-19 helped to affirm the belief that the government was only using vulnerable communities to make money. A study conducted by Dryhurst et al. [13] in ten countries across Europe, America, and Asia found the contrary that people who had direct personal experience with the virus perceived greater risk compared to those who had not experienced a direct encounter. This was motivated by respondents' trust in science, medical practitioners, and their knowledge of COVID-19. In the case of Ghana, it is not surprising that distrust of the healthcare system was high, as this was influenced by the negative attitude of health workers.

Family and community values are derogatory to speak ill of the dead and it is shameful for a family member to die from COVID-19-related causes which contributed to denial among persons whose relatives died from COVID-19. Similarly, the fear of stigmatization among family members about having a relative recovering from diseases such as COVID-19 and HIV/AIDS has been noted in other studies [42, 43]. Bandoh et al. [43] noted that even those who tested positive for COVID-19 were unwilling to isolate for fear of stigmatization on them and their family members. Lawal and Adeola [44] also reported that stigmatization contributed to the residents of the Limpopo Province in South Africa, raising concerns about relocating persons arriving from Wuhan, China, for quarantine in the province.

Misconceptions about COVID-19 such as the belief that Africa is too hot for the virus to thrive, coughing is a sign of COVID-19 even without a laboratory test, the virus is a punishment from the supreme being, and it is simply catarrh contributed to disbelief and poor attitudes towards COVID-19 prevention measures. Misconceptions about COVID-19 and myths are not surprising considering the surge in infordemics throughout the world and the ease of access to such information on social media. Unfortunately, misconceptions can influence perceptions and attitudes toward COVID-19 preventive efforts [37]. Another study in Uganda noted the high level of misconceptions about COVID-19, such as some males believing that COVID-19 is a disease that infects only whites [28]. Other studies in Nigeria and Pakistan also reported that there were myths regarding the pandemic, as the majority of respondents believed that the pandemic was God's punishment to humans [18, 45].

Socio-cultural and individual factors such as low literacy level in the study areas, socio-cultural norms, and religious beliefs were found to contribute to the failure to adhere to some of the COVID-19 preventive measures. The socio-cultural norm of reciprocity of having to attend communal functions such as funerals and the fear of being ostracized for observing COVID-19 prevention protocols contributed to the flouting of the measures. Similarly, studies carried out in Iran noted that community members flouted COVID-19 restrictions and failed to observe preventive measures, because of socio-cultural factors such as strong kinship bonds that compelled people to attend social and family functions, which made it impossible for them to adhere to social distancing and other preventive measures [46, 47]. Also, a study in Gambia reported that community members were unable to adhere to COVID-19 prevention measures, due to poverty and social norms such as the need to attend social functions, visit the market, and shaking hands with friends as a sign of respect and other forms of socialization [48].

Poverty and unemployment also contributed to the inability of community members to afford COVID-19 preventive items as several respondents indicated that they did not have money to buy nose masks and soap to facilitate COVID-19 preventive practices. This is not surprising, because most of the respondents worked in the informal economy and lived from hand to mouth before the advent of COVID-19. Thus, they were no longer earning money when the COVID-19 restrictive measures were instituted by the Ghanaian state. Besides, they did not have any savings to fall on. A study in Burkina Faso reported that COVID-19 had had a drastic effect on rural and urban households, increasing their vulnerability and contributing to food insecurity, so the priority for them was to find food rather than protective items to combat the pandemic [49]. Similar studies carried out in poor communities in Ghana and South Africa suggest that such communities failed to cooperate with governmental COVID-19 regulations, because of the lack of infrastructure and poverty rather than an unwillingness to engage in behavioral change [20]. This study revealed that the Ghanaian government's relaxation of the COVID-19 preventive measures misled community members to believe that COVID-19 had been eradicated from the country or the cases had reduced and Ghanaians were no longer at risk of infection. This development motivated a return to large social functions in the two study communities. This put pressure on community members who had previously tried to observe COVID-19 preventive measures. Others started using nose masks selectively and hugging one another, thus flouting COVID-19 preventive measures. It is thus not surprising that the number of COVID-19 cases in Ghana with the advent of the delta variant has surged, resulting in the government reinforcing some of the preventive measures [6]. Similarly, Feyisa [15] found that people who live in settings where restrictions on movement have been eased have a high tendency of not adhering to COVID-19 protocols compared to those who live in settings that were still experiencing lockdown.

This study has several strengths. Important themes and concepts to the literature on COVID-19 such as trust, motivation, demotivation, community values, myths, misconceptions, and poverty have been discussed in depth in this study. While some of the themes and concepts have been featured in other studies [14, 20, 37, 40], their interactions and outcomes have not been explored in depth in those studies.

The study makes important conceptual and empirical contributions to the study of COVID-19 by using focused ethnography to demonstrate the links between public policies, the government's mitigation efforts, and contextual factors, and how they shape attitudes, behavior, and response to a pandemic in vulnerable populations. In addition, the study makes an important methodological contribution to studies on ethnography in the era of pandemics. It has demonstrated how ethnographic studies can support a comprehensive understanding of the interaction between policy implementation, vulnerability, and contextual factors and the consequent effects on vulnerable communities. This is especially very important, because of the nature of COVID-19. The pandemic poses dilemmas and methodological and ethical challenges as well as risks associated with the use of ethnography, considering that the mode of infection is largely through human interaction, which is also a key feature for successful ethnography [50]. Nevertheless, this ethnographic approach enabled the interrogation of vulnerable cultural groups in their natural setting over a period of time, which enabled the study to experience and report on their lived realities [22, 23, 29].

Despite the challenging circumstances of COVID-19 and doing ethnography in such an era, this study was rigorously undertaken, thus positioning it as a harbinger for other studies in ethnography in similar fields.

## 5. Conclusion

This ethnographic study found that the Ghanaian state's approach to dealing with COVID-19 intervention measures, health system, socio-cultural factors, poverty, and existing gaps in infrastructure and social amenities influenced individuals' and communities' perceptions, attitudes, and observance of COVID-19 preventive measures. It is therefore critical that future interventions take into consideration the role of the community in the process.

The government needs to be consistent with the information it provides to the citizenry. COVID-19 issues tend to be dynamic, which affects the type of information that should be communicated. Additionally, frequent and transparent communication with the citizenry can help build trust in the state's intervention measures. The development of trust would contribute to individuals being motivated to independently adhere to COVID-19 preventive measures. Furthermore, there is a need to involve community leaders, especially for migrant communities in key interventions in their communities. Also, the government needs to engage the communities more to understand their felt needs so that more community-friendly interventions can be initiated to support vulnerable communities.

Health facilities must follow appropriate protocols for testing and tracing of persons who come into contact with COVID-19-positive persons, especially persons who lose their relatives due to COVID-19. In addition, health facilities need to communicate more effectively with families whose relatives test positive for COVID-19 and those who die from COVID-19-related causes. Such an approach will help to build trusting relations with communities for effective collaboration.

COVID-19 and its impact on poor communities are an eye-opener to the Ghanaian government. There is a need that efforts are put in place to develop social amenities in vulnerable communities in the near future to ensure that community members live with dignity and are well prepared to deal with future pandemics of that nature.

### 5.1. Limitations of the Study

This study was conducted in two communities and for that matter cannot be generalized. The study, as is typical of qualitative research studies, focused on a purposive sampling of a limited number of respondents, who shared their subjective experiences, thus, it cannot be generalized. Another limitation of the study is that social media platforms such as WhatsApp and Facebook were not included as means via which community members received information about COVID-19.

## Figures and Tables

**Table 1:** Qualitative data collection methods and category of study respondents.

Category of respondents who participated in IDIs	Study areas	
	Oforikrom Municipality	Adaklu district
*Category*	*No.*	*No.*
Assembly members	1	1
Chiefs and queen mothers	2	2
Community elders	2	2
The staff of the Ministry of Gender, Children, and Social Protection	1	1
GHS officials	2	2
Religious leaders	2	2
Herbalists	2	2
Persons who had been infected or a relative had been with COVID-19	3	3
Persons with disability	3	3
Persons caring for persons with disabilities	3	3
Community volunteers	2	2
Total number of interviews	**23**	**23**

*List of participants for FGDs*		
Women (1, women under 30 years; 1, women over 30 years)	2	2
Men (1, men under 30 years; 1, men over 30 years)	2	2
Total number of FGDs	**4**	**4**

## Data Availability

The data used for this study are available at the University of Health and Allied Sciences repository and can be accessed upon request.

## Supplementary Materials

Supplementary Materials Appendix I, Appendix II, and Appendix III.
