# Supplementary material for: An Ethnographic Study of Multiple Factors Influencing Perceptions, Attitudes, and Observance of COVID-19 Preventive Measures among Rural and Urban Slum Dwellers in Ghana
Source: J Environ Public Health. 2023 Jan 31;2023:1598483. doi: 10.1155/2023/1598483 (PMC9904884; doi:10.1155/2023/1598483)
Supplement: Supplementary Materials — Appendix I, Appendix II, and Appendix III. [file 1598483.f1.zip › Appendix I.docx]

# Appendix I

**University of Health and Allied Sciences**

**Institute of Health Research**

**PMB 31, Sakode-Lokoe, Ho, Volta Region, Ghana**

**Study on socio-economic and health effects of COVID-19 among vulnerable populations: Evidence from the Ashanti and Volta Regions of Ghana**

**IDI guide for community members**

**Section 1: Background characteristics of Respondent**

Community name, age, sex, education, occupation, religious background, tribe/ethnicity

**Section 2: Knowledge on COVID-19 and preventive measures**

1. What do you know about COVID-19?
2. Where did you learn about it?
   1. Who informed you about COVID-19?
   2. What did your informant say about it?
3. How do community members protect themselves from contracting COVID-19?
4. How do you protect yourself from getting infected with COVID-19?
   1. What about your household/compound members?
   2. Who taught you to prevent it?
5. What is the community’s perception about COVID-19?
6. What is your perception about COVID-19?

**Section 3: COVID-19 intervention measures’ effect on economic wellbeing of community members**

1. What work were you doing before the advent of COVID-19?
2. How has COVID-19 affected your work?
   1. Probe for returns in terms of income, customers and workload
3. What have you been doing to ensure that you are able to retain your work?
4. How are you able to take care of your basic needs now that there is COVID-19?
5. Who are the people supporting you to meet your basic needs?
6. How are you able to take care of your dependents?
7. How have all the issues that you mentioned changed from the time you heard of COVID-19 and now?

**Section 4: COVID-19’s influence on the socio-cultural aspects of community life**

1. How has COVID-19 affected interactions in your household?
   1. What about your interactions with people outside your household/compound?
   2. What about community relations?
2. Who has been supporting community members to fight COVID-19?
   1. Probe for government (GHS, NCCE, GES) support and the nature of the support (information, economic, social, spiritual)
   2. How often do you receive such support?
   3. Others such as CHAG, NGOs. What is the nature of support? How often is it provided?
3. How are community leaders helping community members to prevent themselves from getting infected with COVID-19? Probe the following:
   1. What forms of support are they offering community members?
   2. How often do you receive such support?
4. What were community members doing to take care of their health prior to the advent of COVID-19? Probe for where they used to seek health care.
5. How has COVID-19 affected community members’ health seeking behaviour?
   1. Why do you think it has affected community members’ ability to seek health care? (If it hasn’t affected, ask why)
6. What about you, how has COVID-19 influenced your health care seeking behavior?

**Section 5: Coping strategies and recommendations**

1. What do you think community members need most in this time of COVID-19? (Probe for economic, health, social and religious needs).
   1. Pick each need mentioned and ask why it is most needed.
   2. What do you think you need in this time of COVID-19?
   3. Why do you think you need that?
2. How has COVID-19 changed your life?
   1. Probe for positive and negative aspects, social relations, economic, spiritual.
3. What have you learned from COVID-19? (Probe for social, religious, economic)
4. How can you be supported to prevent COVID-19 in your household and compound?
5. In what ways can the government support you with the right information to help you protect yourself and your family from COVID-19?
   1. What about your community?
6. How can community leaders support community members to protect themselves from COVID-19?
7. What about you, what can you do to improve your efforts to prevent yourself and others from contracting COVID-19?
8. We welcome any other recommendations that you would want to share with us regarding COVID-19.
9. Any other comments on COVID-19 are welcome!

**Thank you very much for participating in this interview.**
