# Supplementary material for: An Ethnographic Study of Multiple Factors Influencing Perceptions, Attitudes, and Observance of COVID-19 Preventive Measures among Rural and Urban Slum Dwellers in Ghana
Source: J Environ Public Health. 2023 Jan 31;2023:1598483. doi: 10.1155/2023/1598483 (PMC9904884; doi:10.1155/2023/1598483)
Supplement: Supplementary Materials — Appendix I, Appendix II, and Appendix III. [file 1598483.f1.zip › Appendix II.docx]

# Appendix II

**University of Health and Allied Sciences**

**Institute of Health Research**

**PMB 31, Sakode-Lokoe, Ho, Volta Region, Ghana**

**Study on socio-economic and health effects of COVID-19 among vulnerable populations: Evidence from the Ashanti and Volta Regions of Ghana study**

**IDI study guide for community leaders (chiefs, queen mothers, herbalists, religious leaders, elders)**

**Section 1: Background characteristics of respondent**

**Community name,** age, sex, education, occupation, position in the community, religious background, ethnicity

**Section 2: Knowledge of COVID-19 and preventive measures**

1. How did community members learn about COVID-19?
   1. Probe for sources such as local government, health institutions, CHAG, NGOs
   2. The channel that the source used to get information across to community members (fliers, radio talks etc.)
   3. Probe for frequency of information received on COVID-19 and content of the information
   4. How are community members using information that they have received from the mentioned sources to protect themselves?
   5. How were you involved in the process?
2. What do you perceive COVID-19 to be? (Probe for economic, religious, social, political, biomedical etc.)
   1. What do community members perceive COVID-19 to be?
3. How are community members protecting themselves from COVID-19?
4. How did community members learn to prevent it?
5. Prior to government’s support what were you doing to protect yourself from COVID-19?
   1. What about the community?
6. What support have you received from the government (GES, GHS, NCCE etc.) in your efforts to prevent COVID-19?
   1. Probe for NGOs
   2. Probe for community efforts, churches etc.

**Section 3: Most Vulnerable groups in communities**

1. Who are the most vulnerable in this community in terms of the ability to prevent themselves from contracting COVID-19?
   1. Probe for people who depend on others i.e., new born babies, disabled, mentally challenged, persons with disease conditions, pregnant women, the aged)
2. How have persons with disabilities been supported to prevent themselves from being infected with COVID-19?
   1. Probe for forms of support provided.
   2. Who provides the support?

**Section 4: Communities as partners**

1. What needs are the most important for you to be able to prevent yourself from getting COVID-19?
   1. What about the community?
   2. Probe for food, employment WASH, PPEs, health care etc.
   3. Probe for why each need mentioned is important for preventing COVID-19?
2. Which external bodies have been supporting your community in the fight against COVID-19?
3. What is the form of support that they have been offering you? (Probe for services offered, donation, information etc.)
   1. By what means/channels do they provide the support?
4. How are you involved in the process of providing support to your community?
5. How are you supporting your community in the prevention of the spread of COVID-19?

**Section 5: Communities owning the transformation process**

1. How has COVID-19 changed your life?
   1. Probe for positive and negative aspects, social relations, economic, spiritual.
2. What have you learned from COVID-19? (Probe for social, religious, economic)
3. How do you think you and the rest of your community members can continue to protect yourselves from COVID-19 and other infections?
4. What are your community’s strategies to protecting community members from getting COVID-19? (Probe for traditional, modern, for each strategy mentioned ask what it entails)
5. How do you think you can protect yourself from such future occurrences?

**Community health workers and volunteers**

1. What community health groups exist in this community?
2. How do they support the community in the fight against COVID-19?
3. What forms of support do they provide?

**Recommendations**

1. What do you think community members need most in times of COVID-19? Probe for economic, health, social and religious needs.
   1. Pick each need mentioned and ask why it is most needed.
   2. What do you think you personally need in this time of COVID-19?
   3. Why do you think you need that?
2. What do you think is the best way for the government (district assembly, GES, GHS etc) to engage with your community on COVID-19 and other related issues?
   1. Probe for other institutions, NGOs, CHAG/ faith-based
   2. How should community leaders be involved?
3. What do you think can be done to improve information and education on COVID-19 in your community?
   1. How can external bodies engage with you well on such matters?
   2. How can community leaders contribute to such a process?
   3. What about community members, how will community members be able to support such a process?
4. What other ways can government support you to fight COVID-19?
5. Any other comments and recommendations

**The interview has ended. Thank you very much for participating**
