# Supplementary material for: An Ethnographic Study of Multiple Factors Influencing Perceptions, Attitudes, and Observance of COVID-19 Preventive Measures among Rural and Urban Slum Dwellers in Ghana
Source: J Environ Public Health. 2023 Jan 31;2023:1598483. doi: 10.1155/2023/1598483 (PMC9904884; doi:10.1155/2023/1598483)
Supplement: Supplementary Materials — Appendix I, Appendix II, and Appendix III. [file 1598483.f1.zip › Appendix III.docx]

## **Appendix III: Observation checklist**

**University of Health and Allied Sciences**

**Institute of Health Research**

**PMB 31, Sakode-Lokoe, Ho, Volta Region, Ghana**

**Study on socio-economic and health effects of COVID-19 among vulnerable populations: Evidence from the Ashanti and Volta Regions of Ghana study**

**Part 1**

Transect walk to find out important locations in the community and for familiarization (Community centres – play COVID-19 messages and announcements)

**Part 2**

**Community preparedness**

- Are there hand washing infrastructure (tippy taps, veronica bucket, soap, water must be present) dotted around the community?
- Are there posters on COVID-19 pasted in public places?
- Where do community members hang out?
- What are interactions in communities about?

**Interpersonal interactions**

- How are people interacting in the communities?
- Are people using the protocols such as:
  - Nose mask
- Washing of hands
- Using sanitizers
- Observing social distance

**Household level**

- What is the medium of communication on national issues?
- Do households own radios, TVs etc, smart phones?
- Do they listen to radio talks?
- What times are the radio talks aired?
  - Who listens to them?
- Are there persons with disabilities in households?
- Who communicates COVID-19 information to persons with disability (blind, deaf, crippled)
- How are they being protected from contracting COVID-19?

**Researcher**

- Observe and document how communities treat you as you go around with your mask
- Observe how they interact with you
